# Compressive strength assessment of concrete with brick chips using the CAPO-test

**DOI:** 10.1038/s41598-024-59560-z

**Published:** 2024-06-05

**Authors:** Umme Suraya Nasrin, Abul Khair, Raquib Ahsan

**Affiliations:** Department of Civil Engineering, Bangladesh University of Engineering and Technology (BUET), Dhaka, 1000 Bangladesh

**Keywords:** CAPO-test, Compressive strength assessment, Brick aggregate, In-situ concrete strength, Structural assessment, Civil engineering, Engineering

## Abstract

This study presents the findings of an extensive investigation to evaluate the precision and reliability of a non-destructive CAPO-Test in estimating the in situ compressive strength of concrete made from machine-crushed brick aggregates. To estimate the compressive strength of concrete built from brick and stone aggregates for several target strengths at various ages, the CAPO-Test, core test, and standard cylinder test were used. The results of the core test and cylinder test are correlated with the CAPO pullout force, demonstrating a strong relationship between pullout force and compressive strength. The results suggest that the CAPO-Test displays estimated strengths ranging from 5 to 17% of the cylinder strength and 0–6% of the core strength for concrete containing brick chips. The study validates the CAPO-Test's reliability in conducting in-situ concrete strength assessments.

## Introduction

There is typically a discrepancy between the strength of concrete in structural applications and the strength exhibited by control specimens and the fact that structural concrete is transported, compacted, placed, and cured differently than laboratory concrete cast in cylinders or cubes. To circumvent such constraints, significant efforts have been made to create alternative testing methods, notably non-destructive nature, that would allow an assessment of the quality of concrete and its behaviour in the structure.

Various methods have been proposed for assessing the compressive strength of concrete in its applications. One of these methods acceptable to many researchers^1–5^, is pullout testing of concrete, which can be broken down into two main types: the LOK-TEST, which employs a disc inserted into newly mixed concrete, and the CAPO-Test, which employs an enlarged ring placed in an undercut recess in the hardened concrete^4,6^. The CAPO-Test method is often referred to as a Non-Destructive Test (NDT) in various situations due to its ability to cause minimum damage to the concrete surface without affecting its structural integrity^7–9^.

The evaluation of in-situ concrete compressive strength has significant importance for several purposes. These include the maintenance of quality control in new building projects, the resolution of contractual disputes, the assurance of structural capacity for altered use, and the mitigation of damage caused by fire, fatigue, overload, or environmental variables. The existing methodologies entail the development of specific conversion models by utilizing both destructive and non-destructive testing techniques. The CAPO-Test, which is a non-destructive technique, is used to estimate the strength of concrete at specific test areas^11^. Although pull-out (CAPO-Test) tests are limited to the external concrete layer of structures, they have been proven to be a reliable technique for evaluating the in-place strength of concrete^2^. Pull-out tests are considered to be one of the most reliable non-destructive methods for measuring the in-place compressive strength of concrete^12^.

The CAPO-TEST is an indirect technique that requires the application of an empirical correlation to ascertain the in-situ compressive strength of concrete, relying on the parameter measured by the test method^1^. The CAPO-Test has demonstrated a notable relationship with compressive strength. Analysing the state of stresses in the pull-out test poses challenges, and the obtained strength level in this test indicates its potential for assessing the direct shear strength of concrete^13^. The study conducted by Jensen and Braestrup^14^ showcased the application of Coulomb's criterion to analyse sliding failure. Their findings revealed a clear correlation between the pull-out force and the compressive strength of concrete.

Crushed brick chips (BC) are commonly utilized in concrete constructions in Bangladesh because of the limited availability and high demand for stone chips. The strong demand and restricted availability of stone chip aggregates have resulted in rising prices over time. Consequently, BC has emerged as a widely accepted substitute in the field of concrete construction.. To reduce the cost of manufacturing concrete, BC aggregates are used as an alternative to stone chips and also give a good quality of concrete^15,16^. The investigation of the compressive strength of concrete incorporating brick aggregate is crucial. Therefore, it can be suggested that there is a compelling need to investigate the fundamental structural integrity of BC concrete in the context of brick concrete constructions in Bangladesh. Hence, it is important to conduct an in-depth investigation to gain a deeper understanding of the in-situ compressive strength of concrete containing brick chips.

The study assesses the effectiveness of the CAPO test in determining the strength of concrete, specifically when using machine-crushed brick aggregates. The text emphasizes technological progress, significant associations with conventional testing, and the impact of aggregate types.

The objectives of this study are (a) to assess the variation of concrete CAPO-test strength versus conventional cylinder compressive strength at various ages for concrete with brick chips with varying target strengths; (b) to assess the discrepancy in the compressive strength of concrete specimens containing brick chips, as evaluated by the CAPO-Test and core tests and c) to investigate how concrete with stone chips with different target strengths varies in CAPO-test strength when compared to conventional cylinder compressive strength at various ages.

## Materials and methods

### CAPO-TEST mechanism

The CAPO-Test equipment is utilized to acquire a dependable approximation of the in-situ compressive strength of concrete on existing structures, following the pullout test procedure outlined in ASTM C900-15^17^, BS 1881:207^18^, EN 12504-3^19^, and CSA A23.2-15C^20^. To ensure accurate measurements, the hydraulic pull machine must be equipped with a precision electronic gauge calibrated from 0 to 100 kN. This gauge should possess the capability to store test data, including the peak value attained during testing, as well as the corresponding time and date. The peak value is displayed once the test has been concluded. The gauge has an internal resolution of 0.01 kN; however, the displayed pull force is rounded to the nearest 0.1 kN. The LOK-TEST can utilize the same pull device.

**Figure 1:**
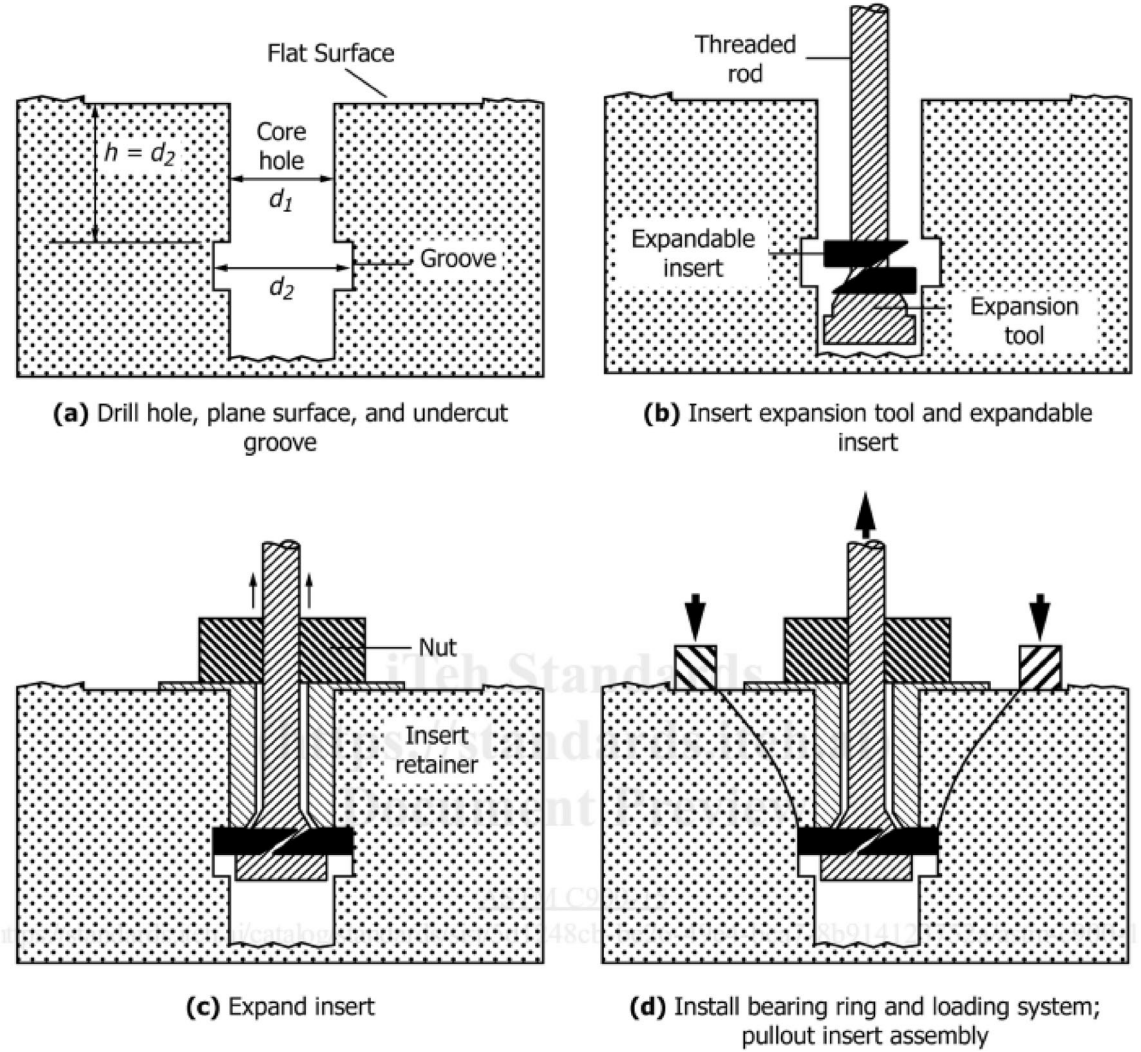
Schematic of Procedure for Post-Installed Pullout Test (after ASTM C900-15^17^).

**Figure 2:**
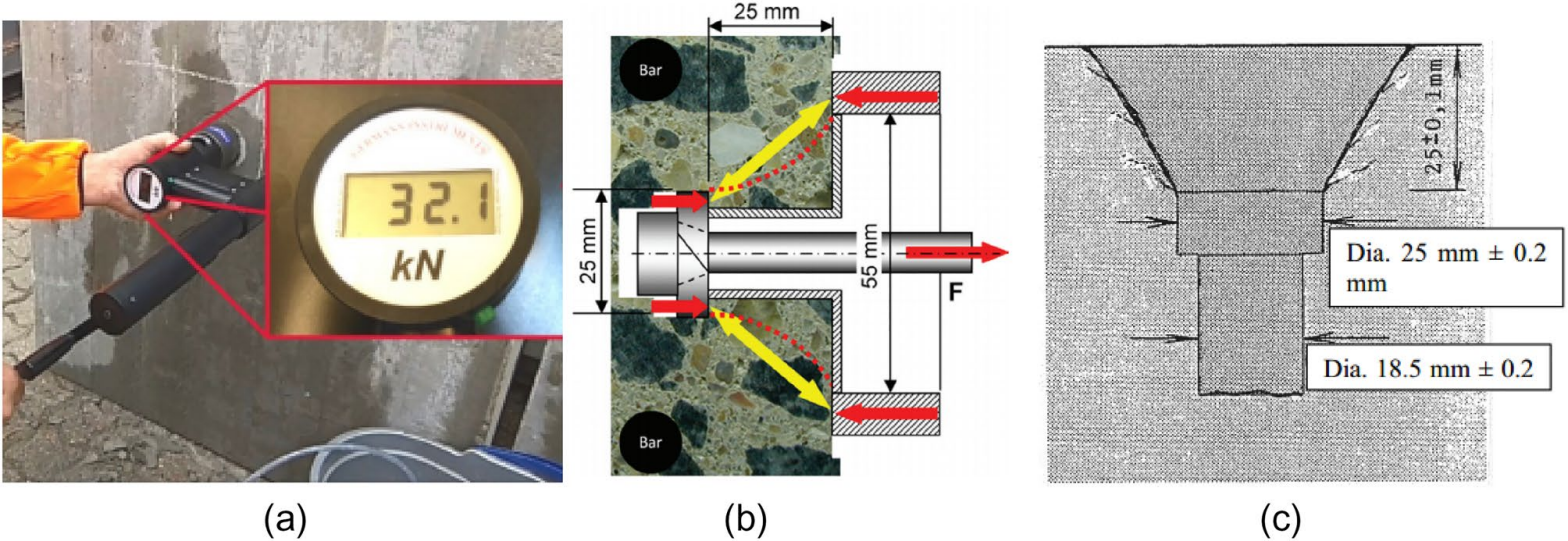
(**a**) The CAPO device from Germann Instruments. (**b**) Cross-sectional view of the CAPO-TEST (**c**) the cross-sectional analysis of Capo-Test failure, specifically focusing on the occurrence of small circumferential cracking (after Germann Instruments, CAPO-Test Manual^22^).

The term "post-installed" belongs to the feature of CAPO-TEST, whereby it does not require the pre-installation of inserts in freshly poured concrete. The recommended set of steps for conducting a post-installed pullout test is outlined in the ASTM C900-15^17^ standard, as depicted in Fig. 1. The assessment may be performed on a pre-existing structure located in any easily accessible location. The technique involved the execution of several steps^2,5,9,21^, the drilling of a 35 mm core to a depth of 28 mm, the utilization of a pullbolt, and the preparation of planed surfaces. The completion of this task required the involvement of three skilled individuals and a significant amount of time. The Capo-Test methodology was devised through the integration of the Lok-Test technique with the structural arrangement of a piston ring used in internal combustion engines. The Capo-Test employed a contrasting principle, whereby a ring was divided using a sideward cut, compressed, and then directed via a central aperture into a groove of 25 mm in width and 25 mm in depth, as depicted in Fig. 2. The system was referred to as CAPO-TEST, also known as the Cut and Pull-Out test.

**Figure 3:**
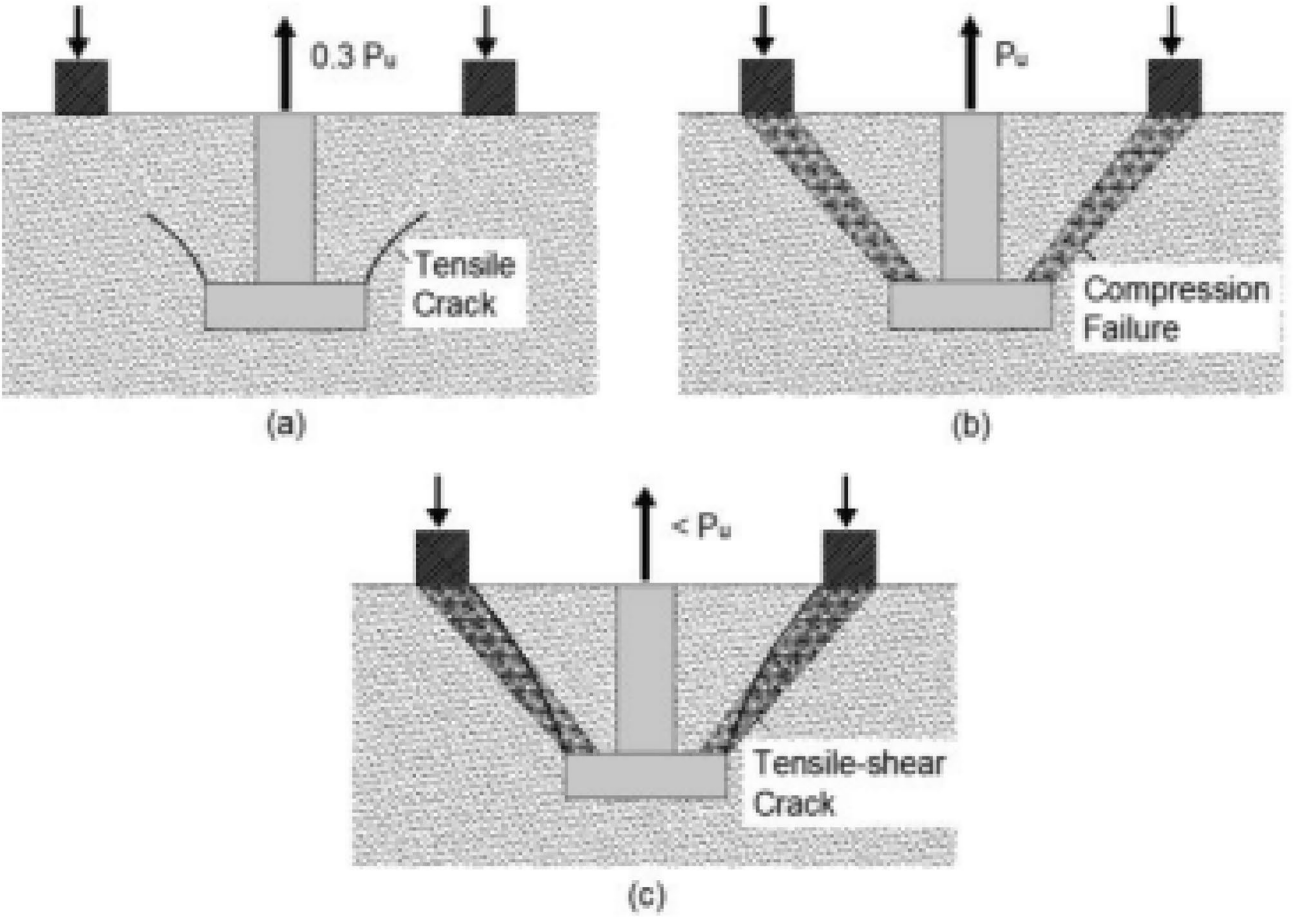
Failure sequence of pull-out tests (after Pannuzzo et al.^9^).

The pullout procedure comprises the utilization of counterpressure, as exemplified in Fig. 1a, leading to the production of compression forces between the expanding ring and the counterpressure. Therefore, the force required for withdrawal functions as a direct measure of compressive strength. The mechanism's failure is attributed to the compression of the concrete within the "strut" that links the disc and the counterpressure^23^. In Fig. 1b, the cross-sectional aspect of the Capo-Test failure is illustrated, revealing the existence of minor circumferential cracking. Additionally, Fig. 3 exhibits the chronological progression of failure seen during the pull-out test. While there exists a significant correlation between the pull-out method and the compressive strength of concrete, a unanimous agreement about the uniaxial compressive failure has not been reached. Consequently, it is necessary to establish a distinct empirical relationship for each particular concrete and testing apparatus.

### Experimental programme

**Figure 4:**
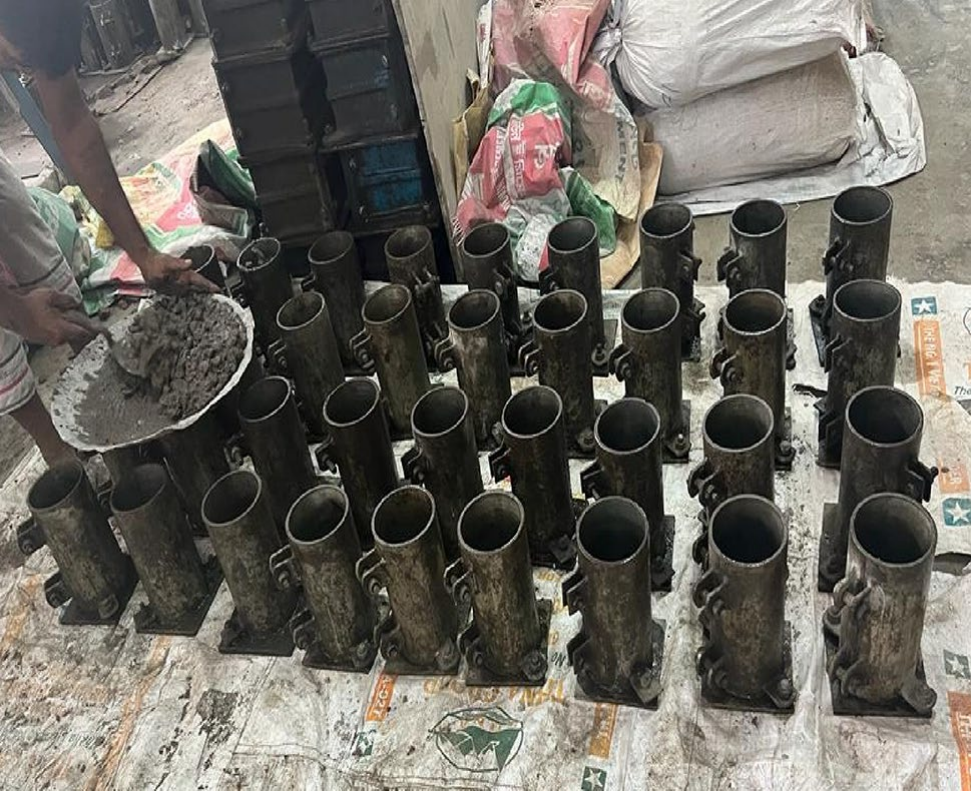
100 mm $\times$ 200 mm Mould Preparation for Cylinder.

**Figure 5:**
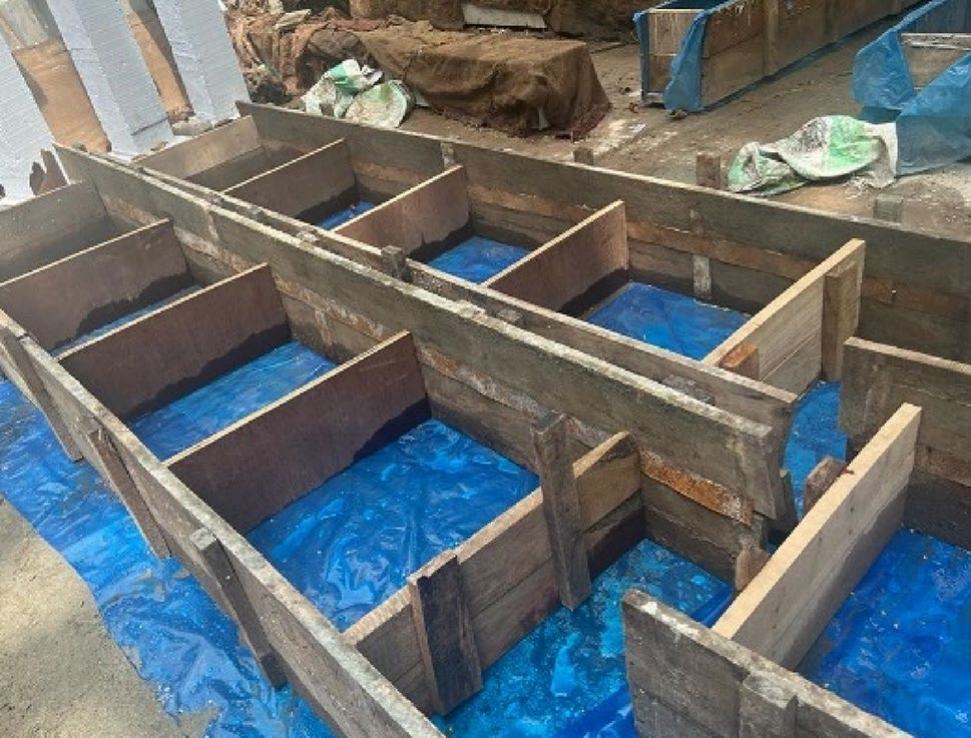
(600 × 600 × 225 mm) Mould preparation for Concrete Blocks.

This study examines many elements that are considered during testing, including the types of tests, the specific age of the test, the different types of aggregates used, the target strength, etc. Three distinct concrete mixes were formulated and subjected to testing at various time intervals, specifically at 14, 28, 56, and 90 days (as depicted in Table 1). According to the ACI 211.1–91^24^ mix design approach, concrete is cast with three target strengths in consideration: 20 MPa, 25 MPa, and 30 MPa (2900psi, 3625psi, and 4351psi). From every batch of concrete mix, twelve (12) standard cylinders measuring 100 × 200 mm (Fig. 4) are cast for each desired strength. A total of 180 cylinders (48 cylinders for each brick aggregate mix) are prepared for testing in the lab, and 15 concrete blocks of 600 × 600 × 225 mm (Fig. 5) in dimensions are cast for the core and CAPO-Tests. Concrete blocks and cylinder specimens are cured for the same amount of time by being covered in wet bags and polythene to keep moisture in. Four (4) CAPO-Tests are conducted from each concrete block in order to get four (4) no. 100 mm x 200 mm cores for the core test.

**Table 1:** Scheme of study.

	Test Type	Course Aggregate Type	Target Strength (MPa)	Number of Specimens/ Points Per Mix	Splits of Concrete age	Total Number Test
Item-I	Cylinder test (100 mm × 200 mm)	Brick chips	20 25 30	12	4	144
		Stone chips	20 25 30	12	1	36
Item-I	CAPO-Test (600 mm × 600 mm x225mm blocks)	Brick chips	20 25 30	4	4	48
		Stone chips	20 25 30	4	1	12
	Core Test (600 mm × 600 mm x225mm blocks)	Brick chips	20 25 30	4	4	48
		Stone chips	20 25 30	4	1	12

## Materials properties

### Particle size distribution

**Figure 6:**
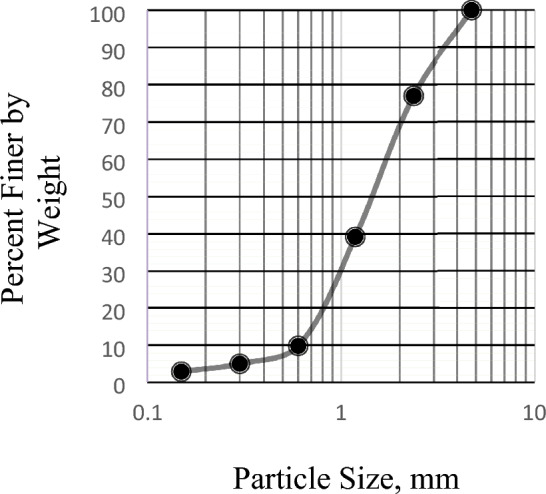
Gradation Curve for BC Aggregates.

**Figure 7:**
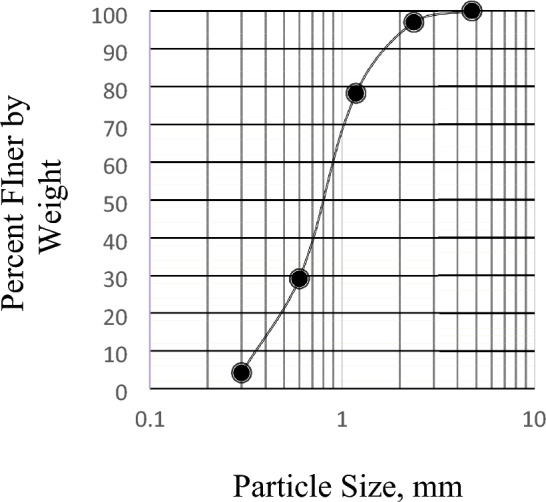
Gradation Curve for Sand.

In order to determine the particle size distribution, a sample of the dry aggregate with known mass was separated through a succession of sieves with progressively smaller openings. The ASTM standard requirements of specification C136/C136M-14^25^ are met by this test procedure. The gradation of aggregate is essential in affecting the workability, strength, and durability of concrete. Figures 6 and 7 display the gradation curves for sand and brick chips, respectively.

### Fineness modulus

The term "fineness modulus" (F.M.) serves as a convenient indicator of the degree of coarseness or fineness shown by a certain material. The test procedure described herein adheres to the specifications given in the ASTM standard C136/C136M-14^25^. The fineness modulus was calculated for both the coarse and fine aggregates, as presented in Table 2.

**Table 2:** Fineness modulus (FM) of coarse and fine aggregates.

Aggregate type	FM
Brick chips	4.66
Stone chips	6.21
Sand	2.90

### Specific gravity and absorption capacity

The determination of the specific gravity (relative density) and absorption capacity of both coarse and fine aggregates is conducted in accordance with the guidelines outlined in ASTM C128-15^26^. The findings are presented in Table 3.

**Table 3:** Specific gravity and absorption capacity of course and fine aggregates.

Type of aggregate	Apparent specific gravity, S_a_	Bulk specific gravity (O-D), S_d_	Bulk specific gravity (S.S.D), S_s_	Absorption capacity (%)
Brick Chips	2.65	1.90	2.18	16.08
Stone chips	2.68	2.51	2.61	1.34
Fine Aggregate	2.32	2.28	2.36	0.73

### Cement properties

The study utilized Ordinary Portland Cement (CEM I Cement). The parameters of the cement are presented in Table 4. The time of setting of hydraulic cement was determined using the Vicat needle method, the compressive strength of hydraulic cement mortars was measured using 2-inch or 50-mm cube specimens, and the fineness of hydraulic cement was assessed using an air-permeability apparatus. These tests were conducted following the standards ASTM C191-13^27^, ASTM C109-13^28^, and ASTM C204-11^29^, respectively.

**Table 4:** The properties of the cement.

Normal consistency (%)	Initial setting time (min	Final setting time (min)	Final setting time (min)	Fineness (m^2^ /kg)
26.5	134	385	296	296

### Mix design for target strength

The specified target strengths for this experimental purpose are 20 MPa (2900 psi), 25 MPa (3625 psi), and 30 MPa (4351 psi). This experiment utilized two distinct categories of coarse particles. There is a total of six mix ratios. The mix design has been conducted following ACI 211.1–91 guidelines. The table provides the mix proportions for concrete built using brick chips, targeting strengths of 20 MPa (2900 psi), 25 MPa (3625 psi), and 30 MPa (4351 psi), as shown in Table 5. This investigation utilized clay-burnt bricks with an average strength of 25.3 MPa for three brick samples prior to their crushing.

**Table 5:** Mix proportion for different target Strength of Concrete using Brick chips.

Materials	Mix proportion for 1.0 m^3^ concrete		
	Target strength, 20 MPa	Target strength, 25 MPa	Target strength, 30 MPa
Water	216 kg	209 kg	200 kg
Cement	360 kg	380 kg	400 kg
Sand (SSD)	770 kg	780 kg	760 kg
Brick (SSD)	880 kg	880 kg	880 kg

### Concrete casting

**Figure 8:**
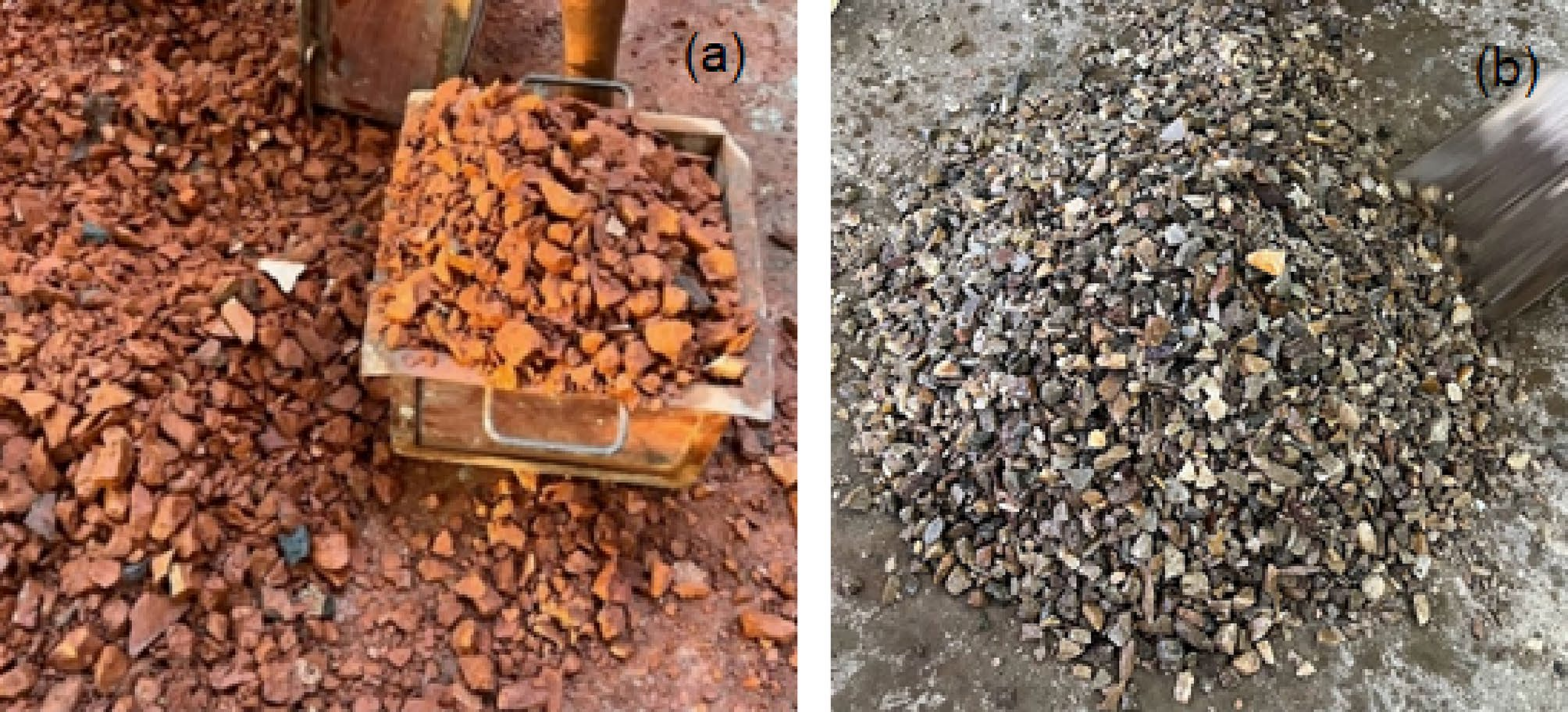
Sprinkling water with Coarse Aggregates, (**a**) brick chips and, (**b**) Stone chips.

The concrete was cast outdoors on a sunny day during the monsoon season, with a temperature of 34 °C and humidity measured at 55.3%, as recorded in the laboratory next to the casting site. The surrounding environment may have impacted the curing process and subsequent strength of the concrete, perhaps leading to variations in the observed results. The aggregate collection process for the mix design involves laboratory procedures wherein the aggregates are moistened with water to achieve a saturated surface dry (SSD) state by the following day during the casting process (see Fig. 8). The mixer has a capacity of approximately 1m^3^ for coarse material. The entire set of materials was partitioned into three separate batches, each maintaining identical proportions. The concrete mixer machine was configured to rotate at a speed of four rotations per minute.

**Figure 9:**
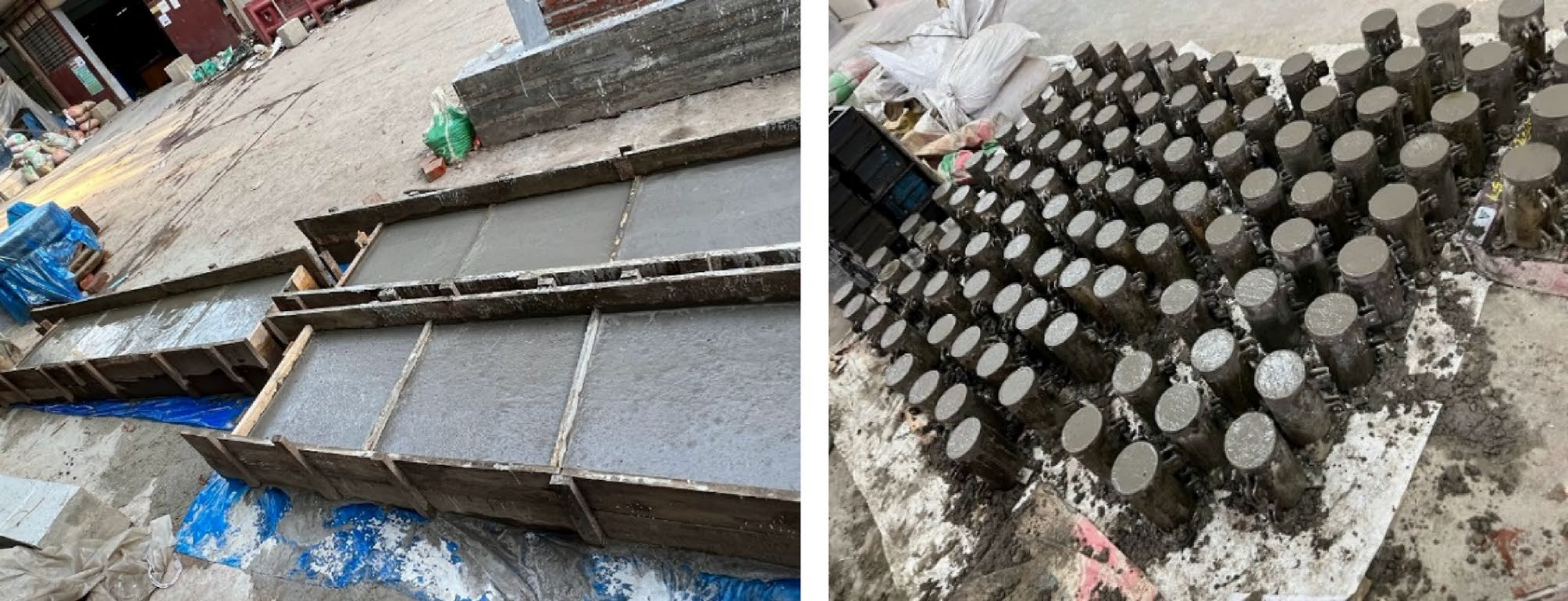
Casting of Concrete in specified mold.

**Figure 10:**
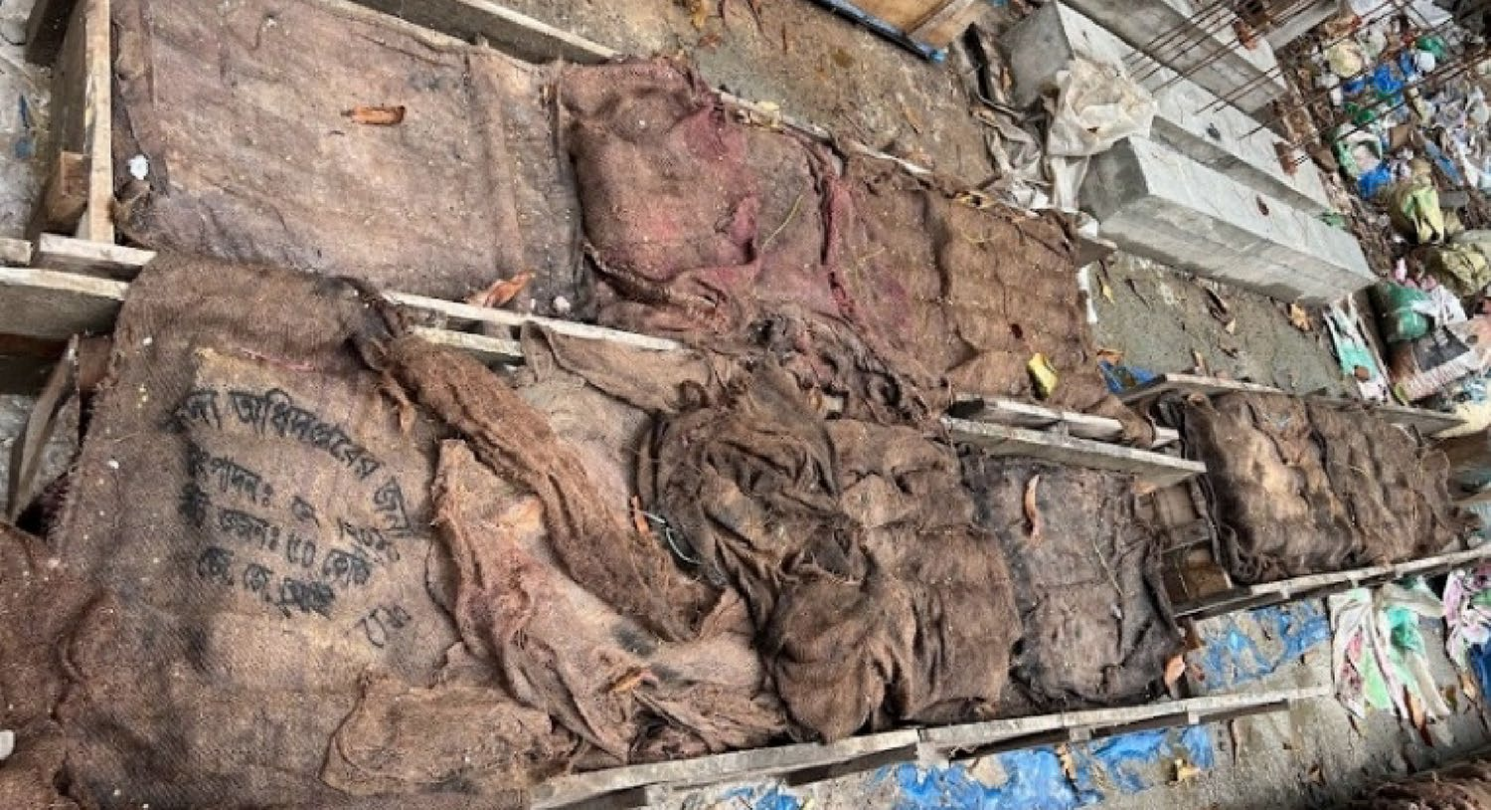
Curing of Concrete Cylinders and Blocks.

Following the necessary modifications to the concrete mixture to address any deviations from the desired slump, the freshly mixed concrete was promptly poured into various molds. It was then compacted using a vibrator (Fig. 9) and subsequently placed in an exposed area for the purpose of curing, as depicted in Fig. 10.

Both the cylinders and concrete block underwent the same curing procedure, as depicted in Fig. 10. Following its removal from the mold, each cube was then coated with damp gunny sacks. In order to mitigate water loss resulting from evaporation, the gunny sacks were enveloped in polythene sheets and securely fastened using ropes. An aperture was positioned on the upper surface to allow for daily water injection, which helps maintain most effective curing conditions. . The strength of concrete has a positive correlation with time, provided that adequate moisture and a suitable temperature are present to facilitate the process of cement hydration. Rapid desiccation of concrete results in significant shrinkage during the initial stages of curing. Insufficient curing is a contributing factor to the development of weakened and dusty surfaces that exhibit subpar resistance to abrasion.

### Human participants

In the discipline of civil engineering, people serve solely as laborers.

### Informed consent

All workers and authors have provided informed consent for the public display of identifying information and photographs in this online open-access article. No concerns have been raised regarding the inclusion of such content.

## Result and discussion

**Figure 11:**
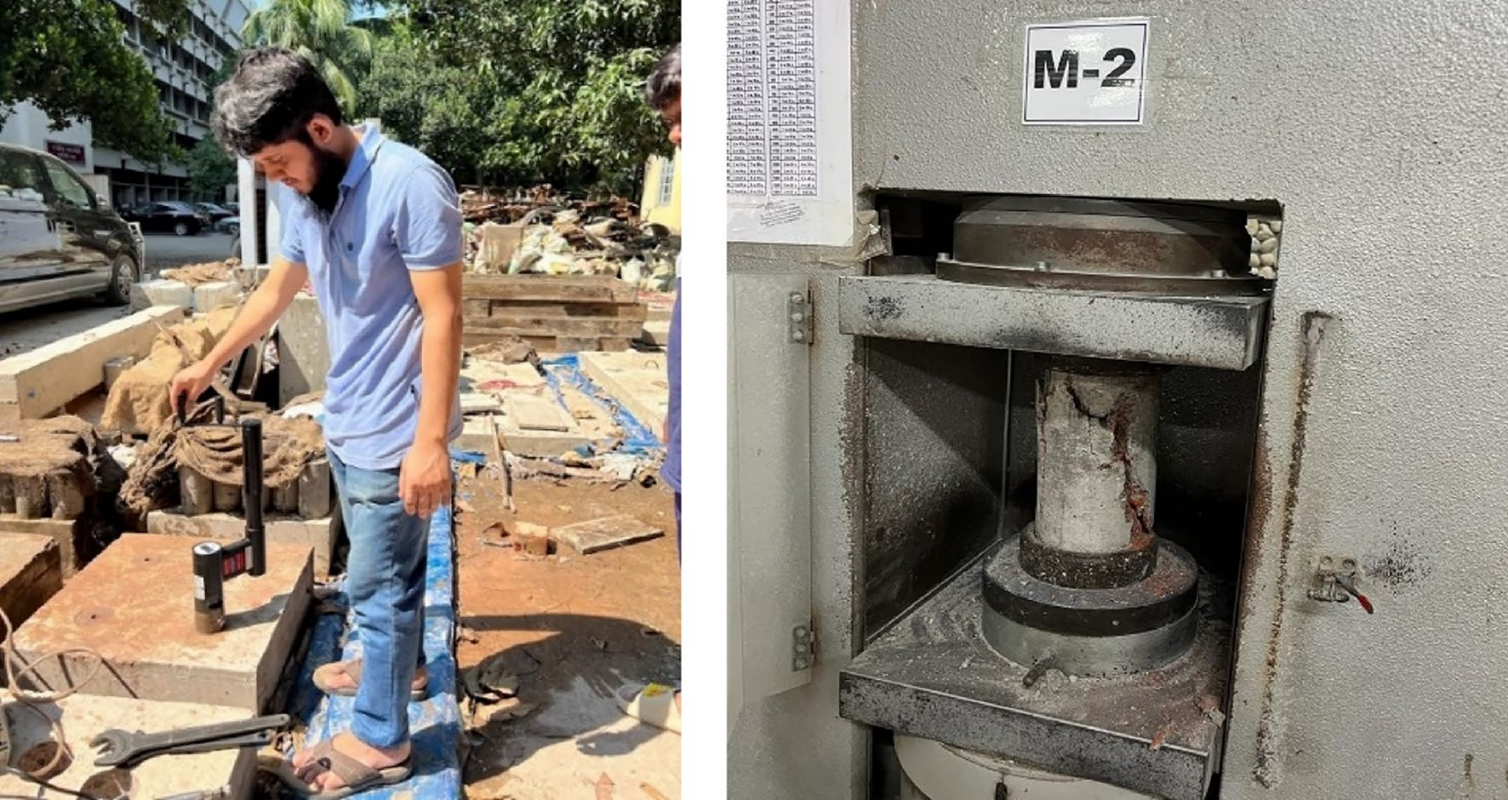
Pull out force measured by CAPO-Test and lab test of core sample.

This paper provides a succinct description of the statistical analysis used in our experimental research, with a focus on Microsoft Excel spreadsheet software. Descriptive statistical measures, such as mean, standard deviation, and correlation coefficients, were used to properly analyze the data, assuring transparency and reproducibility in our results. Following the completion of the curing process on the designated dates of 14, 28, 56, and 90 days, several tests were conducted, including the cylinder test, CAPO-Test, and core test. The procedure for conducting the cylinder test involves subjecting moulded cylinders to an applied axial load, exerted at a controlled pace, until failure occurs. This method is outlined in the ASTM C39/C39M-15a^30^ standard. The CAPO-Test was conducted using a machine made by Germann Instruments A/S in 2015, by the ASTM C900-15^17^ standard. The manufacturer supplied a calibration chart for the particular device. The core collection, preparation, and testing procedures were conducted per the standards ASTM C617/C617M-15^31^ and ASTM C42-18^32^. Figure 11 illustrates the application of a pulling force on a CAPO-Test machine and the measurement of compressive strength on a core sample using Universal testing equipment.

### Compressive strength of concrete against target strength

The concept of target strength in concrete pertains to the predetermined or desired degree of strength that is necessary for a specific application or structural component. It is important to acknowledge that the desired strength generally surpasses the average measured compressive strength in order to accommodate for discrepancies in concrete characteristics, workmanship, and other sources of uncertainty. The presence of a margin of safety serves the purpose of ensuring that the concrete will satisfy the intended performance criteria and exhibit sufficient strength and durability during its service life.

**Figure 12:**
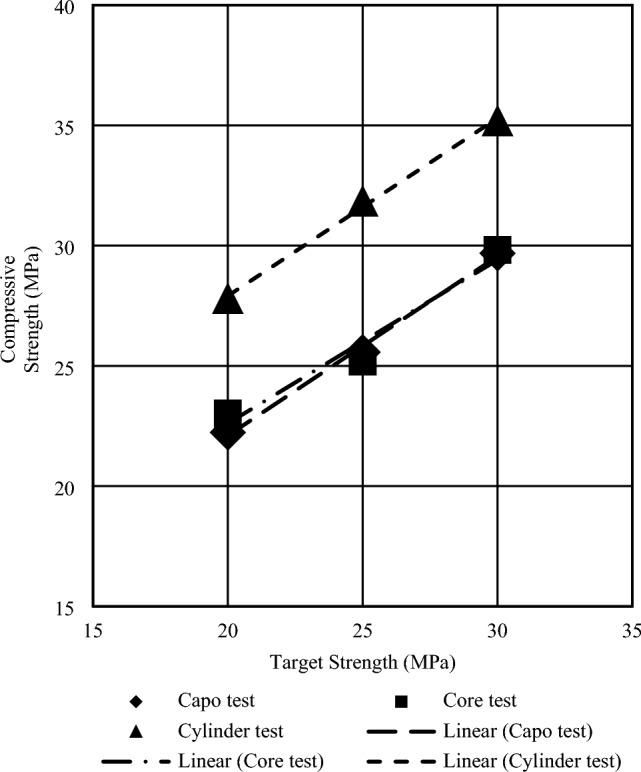
Regression Analysis Computing Compressive Strength to Target Strength for Stone Chips at 28 days.

**Figure 13:**
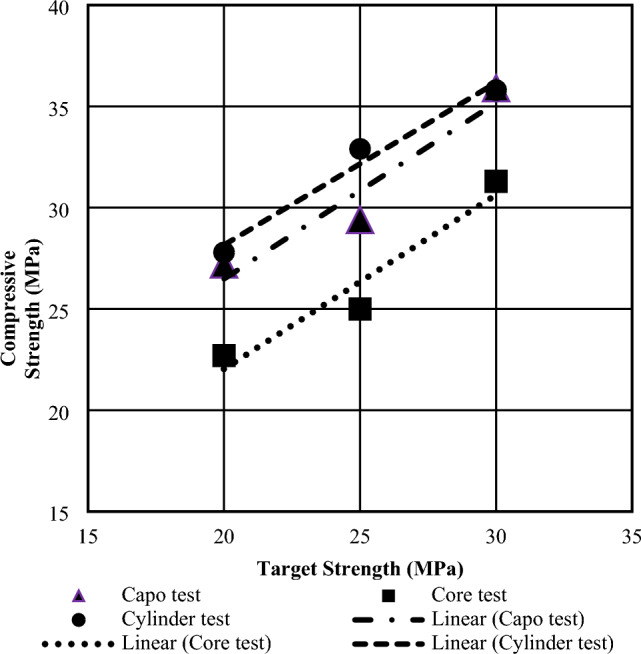
Regression Analysis Computing Compressive Strength to Target Strength for Brick Chips at 28 days.

Figures 12 and 13 display the average compressive strength of concrete samples created using stone chips and brick chips at 28 days, as determined by the cylinder, core, and CAPO-Tests. The position of the CAPO-curve in BC aggregate concrete is significantly affected by the combined effects of water absorption and the duration of the curing process on porosity and strength. This suggests that changes in these factors can have a considerable impact on the overall behavior and properties of the material under investigation. In each of the aforementioned regression equations, it has been shown that the regression coefficient (R^2^) exceeds 0.9 and predominantly falls within the range of 0.94–1.0, hence signifying a strong association between the goal strength and the crushing strength. Figures 12 and 13 also present a comprehensive plot illustrating the relationship between the compressive strength of all specimens and their corresponding target strength. Linear regression analyses were conducted to calculate the compressive strength based on the goal strength, and it was shown that the coefficient of correlation was highest for stone aggregate concrete. The reason behind this is that the ACI mix design is specifically designed for concrete that incorporates stone aggregates.

## CAPO-TEST correlations

**Figure 14:**
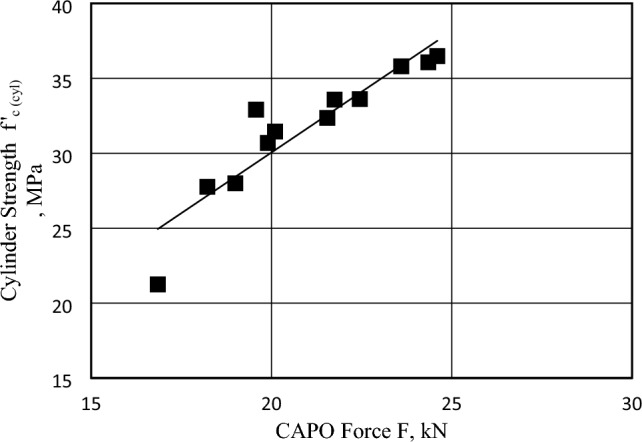
Correlation Between CAPO Force (F) and Cylinder Strength for Concrete Made with Brick Chips.

**Figure 15:**
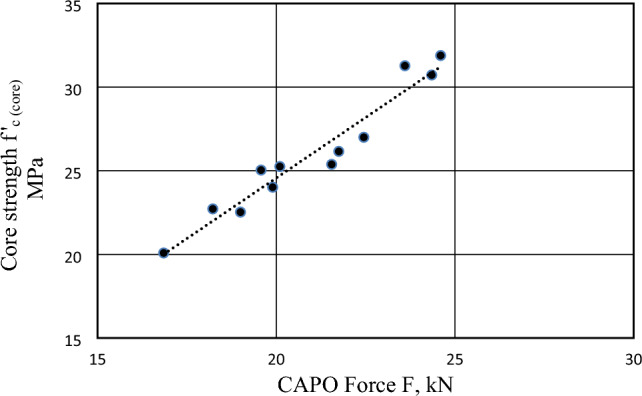
Correlation between CAPO Force (F) and Core Strength for Concrete Made with Brick Chips.

The objective of the "Correlation between CAPO Force and Core and Cylinder Strength" study is to establish a relationship between the non-destructive CAPO-Test and the extracted core strength, as well as the compressive strength of cylindrical specimens in concrete containing brick chips. This study entails the collection of data from CAPO-Testing, as well as the performance of laboratory experiments on extracted cores and cast cylinder specimens to ascertain their strengths. By employing rigorous statistical analysis and conducting thorough comparisons, a correlation model will be constructed estimating to the strength of concrete. This estimation will be based on CAPO force measurements, thereby facilitating dependable evaluations of concrete in its installed state, without the need for destructive testing techniques. This study examines the link between CAPO, core, and cylindrical specimens through the utilization of linear regression models. Regression coefficients are computed to assess the appropriateness of these relationships, as depicted in Figs. 14 and 15. The data indicates a stronger link between the CAPO force and the core strength of concrete compared to the CAPO force and cylinder strength. The linear relationship presented above can be utilized to ascertain the in-situ strength of concrete based on the CAPO-Test.

The present study has examined the relative disparities among the test outcomes and has subsequently organized them in a tabular format, as presented in the following table.

In order to investigate this topic, an analysis was conducted using brick chips of several grades of concrete and the average estimated concrete strength derived from CAPO force, based on the established correlation. Additionally, the determination of core and cylinder strength is conducted based on the analysis of experimental findings. The relative difference is employed for this purpose, and it is defined as follows.

1 $$\frac{{f{\prime}}_{c (CAPO)} - {f{\prime}}_{c (core)} }{ {f{\prime}}_{c (core)}}$$

where the relative difference is between estimated and measured concrete compressive strength. *f*'_c (CAPO)_ is the average estimated compressive strength of CAPO-Test results using the developed correlation and *f'*_*c (core)*_ is the measured average core strength from experimental results. The values of these relative differences for the laboratory test results are shown in Table 6. The average estimated strength by the CAPO-test using the developed correlation is approximately 5% greater than the measured core strength. Also, it is observed that the average estimated strength by the CAPO-Test using the developed correlation is approximately 5 ~ 17% greater than the measured cylinder strength.

**Table 6:** Relative difference of compressive strength of concrete made with brick chips based on CAPO, core and cylinder tests.

Target Strength (MPa)	Age of Concrete (Days)	Average CAPO force per batch (kN)	Average Cylinder strength (MPa)	Core strength, MPa	Develop correlation for cylinder by Average compressive strength (MPa)	Develop correlation for core by Average compressive strength (MPa)	Difference Cylinder to Capo (%)	Difference Cylinder to Core (%)	Difference Core to Capo (%)
20 MPa	14	16.9	21.3	20.1	24.9	20.01	−17	5	0
25 MPa		19.0	27.2	22.5	28.4	23.12	−4	17	−3
30 MPa		21.6	32.4	25.4	32.6	26.81	−1	22	−6
20 MPa	28	18.2	27.8	22.7	27.2	22.00	2	18	3
25 MPa		19.6	32.9	25.0	29.4	23.96	11	24	4
30 MPa		23.6	35.8	31.3	35.9	29.77	0	13	5
20 MPa	56	19.9	30.7	24.0	29.9	24.42	3	22	−2
25 MPa		21.8	33.6	26.2	32.9	27.10	2	22	−4
30 MPa		24.4	36.1	30.7	37.1	30.86	−3	15	0
20 MPa	90	20.1	31.5	25.3	30.2	24.71	4	20	2
25 MPa		22.5	33.6	27.0	34.0	28.11	−1	20	−4
30 MPa		25.9	36.5	31.9	39.6	33.10	−9	13	−4

Table 7 presented above displays the proposed relationship between CAPO force and the compressive strength of concrete based on this study. Tables 8 and 9 displays the percentage disparities found during the assessment of strength utilizing the correlations suggested in the study. This comparison illuminates the efficacy and precision of the suggested correlations in evaluating the material's strength being studied. Examining these disparities offers valuable understanding regarding the dependability and relevance of the suggested techniques for forecasting strength attributes, thereby enlightening subsequent investigations and practical implementations in the field of study. The experimental correlation equation yielded results indicating a range of 0% to 1% variability for core strength and 30% to 35% variability for cylinder strength. Therefore, both equations yield similar results. The experimental equation demonstrates the viability of utilizing CAPO force as a reliable indicator for the estimation of compressive strength.

**Table 7:** Proposed linear and power correlations for CAPO force with cylinder strength made with brick chips in this study.

Aggregate type	Correlation between	Proposed correlation	*R*^2^
Brick Chips (BC)	CAPO Force with Cylinder Strength	Y = 1.6208X − 2.3643 (X- represents CAPO Force)	0.8469
Brick Chips (BC)	CAPO Force with Core Strength	Y = 1.447X − 4.3762 (X- represents CAPO Force)	0.9371

**Table 8:** Summary of compressive strength of concrete based on developed correlation equation with correlation proposed in this study for cylinder.

Target Strength (MPa)	Age of Concrete (Days)	Average CAPO force CAPO per batch (kN)	Developed correlation*—Average Estimated compressive strength (MPa)	Proposed correlation—Average Estimated compressive strength (MPa)	Percentage different (%)
20 MPa	14	16.9	24.95	16.32	35
25 MPa		19.0	28.43	18.67	34
30 MPa		21.6	32.56	21.49	34
20 MPa	28	18.2	27.17	17.82	34
25 MPa		19.6	29.37	19.31	34
30 MPa		23.6	35.89	23.80	34
20 MPa	56	19.9	29.89	19.66	34
25 MPa		21.8	32.89	21.72	34
30 MPa		24.4	37.10	24.64	34
20 MPa	90	20.1	30.21	19.88	34
25 MPa		22.5	34.02	22.50	34
30 MPa		25.9	39.61	26.41	33

*Correlation developed by Malhotra and Carino^33^.

**Table 9:** Summary of compressive strength of concrete based on developed correlation equation with correlation proposed in this study for core.

Target Strength (MPa)	Age of concrete (Days)	Average CAPO force per batch (kN)	Developed correlation* Average Estimated compressive strength (MPa)	Proposed correlation—Average Estimated compressive strength (MPa)	Percentage different (%)
20 MPa	14	16.9	20.01	20.12	−1
25 MPa		19.0	23.12	23.13	0
30 MPa		21.6	26.81	26.77	0
20 MPa	28	18.2	22.00	22.04	0
25 MPa		19.6	23.96	23.95	0
30 MPa		23.6	29.77	29.74	0
20 MPa	56	19.9	24.42	24.41	0
25 MPa		21.8	27.10	27.06	0
30 MPa		24.4	30.86	30.84	0
20 MPa	90	20.1	24.71	24.69	0
25 MPa		22.5	28.11	28.07	0
30 MPa		25.9	33.10	33.13	0

*Correlation developed by Malhotra and Carino^33^.

## Conclusions

The focus of this work is to the evaluation of the in-situ compressive strength of concrete that incorporates brick chips and stone chips, utilizing the CAPO-Test technique. The study's primary findings and conclusions are outlined as follows:

- Studies have shown that the predicted compressive strength of concrete made with stone chips, as calculated by the CAPO-Test, is generally 10–45% less than the strength tested with cylindrical specimens. In contrast, the projected compressive strength of concrete cylinders produced with stone chips typically exceeds the strength determined from core specimens by around 5–20%. These findings are consistent with the general correlations established in previous studies.
- A correlation has been identified that demonstrates a direct relationship between the force exerted by the CAPO device and the compressive strength of the cylinder. The correlation coefficient for the relationship between the age of concrete made with brick chips and the correlation coefficient R^2^ values ranges from 0.81 to 0.99. Additionally, there is a linear relationship between the CAPO force and the compressive strength of cores, with the R^2^ value ranges from 0.95 to 0.99 depending on the age of the concrete made with brick chips.
- A proposed relationship (Y = 1.6208X − 2.3643 for CAPO force and cylinder strength, and Y = 1.447X − 4.3762 for CAPO force and core strength) has been constructed using laboratory test data in order to evaluate the in-situ strength of concrete including brick chips. The equation presented in this study deviates from the developed equation, which is derived from a combination of field and laboratory data including many factors. In contrast, the proposed equation in this study is solely based on laboratory test data.
- The strength of the estimate was determined by employing the established correlation proposed by Malhotra and Carino^33^. The CAPO-Test has revealed that the estimated compressive strengths of concrete with brick chips are around 5 to 17% higher than the values measured using cylindrical specimens, and approximately 0 to 6% higher than the strengths measured using core specimens. The compressive strength of cylinders using brick chips exhibits a slight increase of approximately 0 to 5% in comparison to the core strength of the concrete.
- Research has indicated that the estimated compressive strength derived from the proposed correlation of the CAPO-Test is lower than the strength of cylinders, however the estimated compressive strength derived from the existing correlation for the CAPO-Test is higher than the strength of cylinders for concrete containing brick chips.
- The core tests conducted on concrete samples with stone chips and brick chips reveal that concrete made with stone chips exhibits a strength increase of 8%, 11%, and 3% when compared to concrete manufactured with brick chips, at target strengths of 20 MPa, 25 MPa, and 30 MPa, respectively. Additionally, the cylinder tests conducted on concrete samples with target strengths of 20 MPa, 25 MPa, and 30 MPa reveal that concrete made with stone chips exhibits 3%, 2%, and 4% higher strength, respectively, compared to concrete manufactured with brick chips.
- The probable divergence of the proposed correlations could be attributed to variances in workmanship, despite the operators' CAPO test experience. This emphasizes the significance of addressing elements beyond methodology, such as performer consistency and skill. These kinds of revelations are essential for improving the dependability and applicability of the suggested correlations in practical.

Further study is recommended to establish a reliable correlation for accurately evaluating the in situ compressive strength of concrete with brick chips using the CAPO-Test.

## Data Availability

Data will be made available on reasonable request (Contract Person: Abul Khair, e-mail: akhairce08@gmail.com).
